# Deep tissue localization and sensing using optical microcavity probes

**DOI:** 10.1038/s41467-022-28904-6

**Published:** 2022-03-11

**Authors:** Aljaž Kavčič, Maja Garvas, Matevž Marinčič, Katrin Unger, Anna Maria Coclite, Boris Majaron, Matjaž Humar

**Affiliations:** 1 Department of Condensed Matter Physics, J. Stefan Institute, Jamova 39, SI-1000 Ljubljana, Slovenia; 2 CENN Nanocenter, Jamova 39, SI-1000 Ljubljana, Slovenia; 3 Faculty of Mathematics and Physics, University of Ljubljana, Jadranska 19, SI-1000 Ljubljana, Slovenia; 4 Institute of Solid State Physics, Graz University of Technology, NAWI Graz, Petersgasse 16, 8010 Graz, Austria; 5 Department of Complex Matter, J. Stefan Institute, Jamova 39, SI-1000 Ljubljana, Slovenia

**Keywords:** Microresonators, Imaging and sensing, Biophotonics

## Abstract

Optical microcavities and microlasers were recently introduced as probes inside living cells and tissues. Their main advantages are spectrally narrow emission lines and high sensitivity to the environment. Despite numerous novel methods for optical imaging in strongly scattering biological tissues, imaging at single-cell resolution beyond the ballistic light transport regime remains very challenging. Here, we show that optical microcavity probes embedded inside cells enable three-dimensional localization and tracking of individual cells over extended time periods, as well as sensing of their environment, at depths well beyond the light transport length. This is achieved by utilizing unique spectral features of the whispering-gallery modes, which are unaffected by tissue scattering, absorption, and autofluorescence. In addition, microcavities can be functionalized for simultaneous sensing of various parameters, such as temperature or pH value, which extends their versatility beyond the capabilities of standard fluorescent labels.

## Abstract (web summary)

Optical microcavities are useful as probes in cells due to their narrow emission spectra and high sensitivity to environment. Here, the authors use the unique spectral features of microcavities, which are unaffected by tissue scattering, and show 3D localisation and tracking of cells deep in tissues.

## Introduction

The introduction of optical microscopy represented a key milestone in biological and biomedical research. Imaging of cellular and subsequently also sub-cellular structures became possible, as well as monitoring of their functions. In most biological tissues, however, strong scattering of visible light^1^ limits the use of standard microscopy to depths well below the scattering mean free path *l*_*s*_ = 1/*μ*_*s*_, where *μ*_*s*_ is the scattering coefficient. It depends strongly on the wavelength, getting smaller with shorter wavelength. At larger depths, the contribution of scattering blurs the image and degrades its spatial resolution. Investigation of deeper tissue layers thus requires invasive biopsy and preparation of 5–10 μm thick histological sections.

Numerous optical methods were developed over the past decades to overcome this problem^2–9^. High-resolution techniques, such as confocal microscopy and optical coherence tomography^3–5,10,11^, rely on detection of ballistic (i.e. non-scattered) photons, and are thus inherently limited to depths below one tissue transport length, where the direction of light becomes completely randomized^2^. The transport length is defined as ${l}^{* }=1/\left({\mu }_{a}+{\mu }_{s}(1-g)\right)$, where *μ*_*a*_ is the absorption coefficient and *g* is the scattering anisotropy factor, defined as the average cosine of the scattering angle. Conversely, optical imaging modalities capable of imaging significantly deeper into the tissue, such as diffuse optical tomography^8^, lack single-cell spatial resolution. Wavefront shaping excels in both resolution and depth of penetration, but it is relatively slow and works best in non-dynamic samples^9^, while photoacoustic and ultrasound-assisted imaging^6,7^ require complex experimental setups involving custom ultrasound transducers. Fluorescence imaging is one of the most important imaging modalities in biology, therefore a number of techniques for deep tissue fluorescence imaging were developed. Fluorescence molecular tomography can reach several centimeters deep by detecting diffuse fluorescent light, but does not achieve single-cell resolution^2,12,13^. Mesoscopic fluorescence molecular tomography is able to image a few millimeter thick biological tissues with resolution in the order of 100 μm^14,15^. DOLPHIN, which is based on the hyperspectral and diffuse imaging in near-infrared can detect 100 μm or 1 mm-sized probes through ~2 cm or ~4 cm thick tissue, respectively^16^. A method for the readout of time-varying sources without the need of imaging was also demonstrated^17^. Novel fluorescent probes with longer emission wavelength and higher brightness are developed to aid for deeper imaging^18^.

Microcavities and microlasers manufactured from biological and biocompatible materials for the study of various biological systems have received considerable attention in recent years^19^. Cell tracking^20–23^, biosensing^24–26^, and super-resolution imaging^27^ were demonstrated, but mostly limited to cell cultures. Several studies involving internalization of large, up to 20 μm-sized, particles of different materials and shapes by various cells did not find any significant effects on the cell viability due to the microparticles for periods of up to 10 days^21,28–30^. Among different types of microcavities, whispering gallery mode (WGM) microcavities based on total internal reflection in spherical microparticles, are the most frequently employed for biological applications. WGM microcavities have typically small size, high Q-factors, and are simple to manufacture.

In the present study, we show that the unique properties of WGM microcavities enable their application in deep tissue without affecting their sensing and tracking capabilities. The concept relies on the high Q-factor optical resonances, which can be reliably detected through media with large scattering, absorption, and autofluorescence^20,25,31^. We demonstrate a localization method based on such optical microcavity probes, which enables localization of single cells with the accuracy of 5 μm in the lateral and better than 40 μm depth accuracy in the axial direction at depths up to ~2 l^*^. Simultaneously, the microcavities can be used for cell tagging and tracking, and for measuring different parameters such as the external refractive index, pH, and temperature. Moreover, only standard optical equipment is required, namely a fluorescence microscope and a spectrometer. In the first part of the article, the working principles of the method are presented using the measurements with WGM microcavities beneath artificial optical phantoms, which enable quantitative estimation of the maximum working depth and the localization accuracy. In the second part, as the main result of the study, localization and sensing are performed with microcavities internalized by cells and injected into biological tissues.

## Results

### Identification of the microcavities by unmixing the spectra

**Fig. 1:**
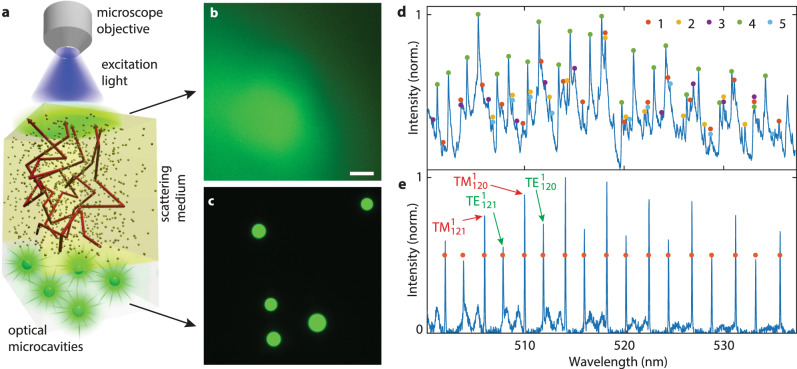
Spectral reconstruction of microcavities beneath a scattering medium. **a** Schematics of the experimental configuration and light propagation. **b** Fluorescence image of the microcavities taken through a 170 μm thick strongly scattering layer. Scale bar, 20 μm. **c** Fluorescence microscope image of the same area without the scattering layer. **d** Spectra taken from the whole area in **b** with the fluorescence background removed. The spectral peaks belonging to a particular microcavity are grouped together. Some peaks are associated with more than one microcavity. **e** Comparison of the spectrum from one particular microcavity without the phantom (blue line) and the reconstructed peak positions (points).

Green fluorescent polystyrene beads with an average diameter of 15 μm were employed as microcavity probes. The size of the microcavities was selected to achieve the desired Q-factor. Below a Q-factor of 3000, the spectral peaks become so wide that there is significant overlap between peaks originating from different microcavities. On the other hand, increasing the Q-factor beyond 10,000 does not bring any advantages, since we are approaching the resolution of our spectrometer. The selected 15 μm microcavities have an average Q-factor of 7000 and are still small enough to enable cellular uptake, which makes them perfectly sized for the purposes of this study. To test the method, a phantom layer mimicking strongly scattering tissue was placed between the microcavities and the microscope objective (Fig. 1a). The phantom had a relatively high scattering coefficient of *μ*_*s*_ = 76 mm^−1^ and *g* = 0.87 at 532 nm resulting in l^*^ = 100 μm. For comparison, in visible light above 450 nm, biological tissues have strong scattering and relatively low absorption, typically resulting in l^*^ ≈ 100–1000 μm and *g* ≈ 0.8–0.9^1^. The locations of the microcavities were completely unrecognizable when imaged through a phantom with optical thickness corresponding to 1.7l^*^ (Fig. 1b, c). However, in the fluorescence emission spectrum collected through the phantom, over 60 peaks could be observed, corresponding to WGMs of 5 different microcavities (Fig. 1d). Because the beads were polydispersed, each microcavity had a unique spectral fingerprint, which could be distinguished as long as their sizes differed by more than ~3 nm. To identify which spectral peaks correspond to each microcavity, an unmixing algorithm was developed (see Methods and Supplementary Fig. 1). The identified peak wavelengths for a given microcavity matched those obtained from the same microcavity without the phantom (Fig. 1e) within an average of 0.014 nm. The microcavity sizes and external refractive indices were calculated by fitting the measured spectral peaks to the characteristic equation for the WGM eigenfrequencies^32,33^. The diameter of one of the microcavities calculated from the reconstructed spectral peaks (Fig. 1e) was 13.0189 μm. For comparison, the diameter of the same microcavity measured without the phantom (13.0186 μm) matched within 0.3 nm. The spectra were successfully detected from microcavities located as deep as 440 μm, corresponding to 3.5 times the transport length l^*^.

The number of microcavities that can be identified from a single spectrum, collected from a particular position on the surface, is limited by the spectral peak overlap. Typically, 5 microcavities were reliably identified. This number was different from case to case depending on how similar were the spectra of microcavities that contributed to the signal. Even just two microcavities can not be identified if they have similar diameters. For the microcavities used here, the probability of two randomly selected microcavities to have a diameter difference of less than 3 nm is 8 × 10^−4^. This probability is quite low, so this case was actually never observed in the experiments. Based on the width of the spectral peaks compared to the free spectral range, the theoretically maximum number of microcavities, which can be identified from a single spectrum, is ~10.

The collection spot can be moved across the sample surface to identify a much larger number of microcavities. As an example, for a typical sample with optical thickness of 2l^*^, the light distribution width at the surface for a single microcavity is 400 μm. Therefore the collection spot should be moved at least this distance to collect light from a different set of microcavities. In practice, however, when moving across the sample the spectral peaks from different microcavities are differently prominent in various positions across this smeared region. This enables identification of different microcavities if moving only by approximately half of the light distribution width, 200 μm in this case. For a typical scanning area of 3 mm × 3 mm and based on the fact that we can reliably identify 5 microcavities from an area of 200 μm in diameter, the theoretical maximum number of resolvable microcavities is ~1400.

The maximum number of identifiable microcavities from a single spectrum could be increased in future by minimizing the peak overlap between different microcavities. This could be achieved in two ways. First, a mixture of microcavities doped with different fluorescent dyes could be used. For example, a blue, a green, and a red microcavity can easily be separated by using three spectral filters, even if they all have exactly the same diameter. Second, the peak overlap could be reduced by decreasing the number of spectral peaks. It has been shown that by using single-mode emission, such as in the case of microdisc lasers^20^, as many as 400 microcavities could be resolved.

### Spatial localization of the microcavities

**Fig. 2:**
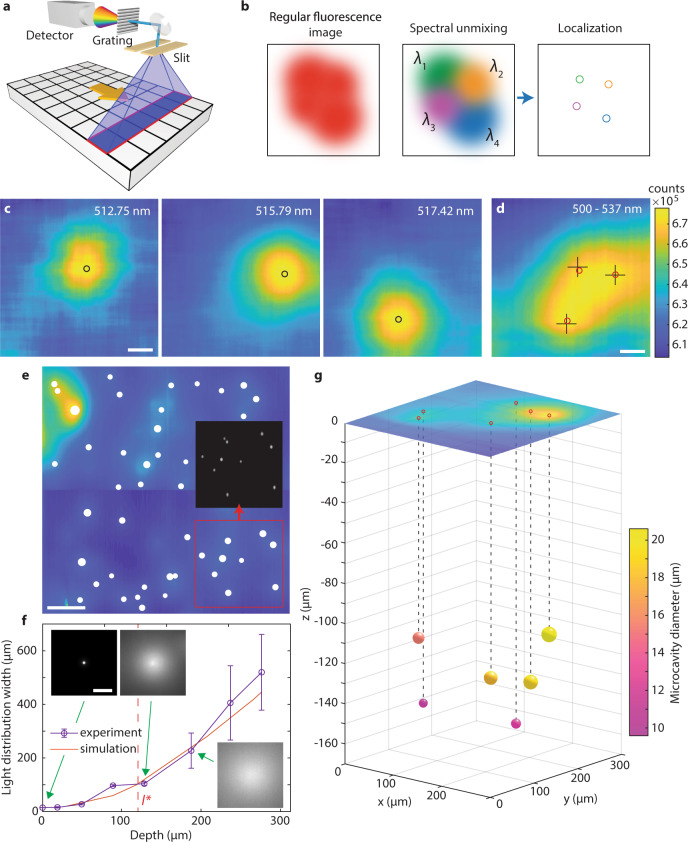
Localization of the microcavities. **a** Schematics of the pushbroom hyperspectral imaging. **b** Principle of the spectral localization. **c** Intensity images acquired through a phantom (1.7l^*^) at three wavelengths, corresponding to WGM peaks from different microcavities. The circles indicate the reconstructed locations. Scale bar, 50 μm. **d** Intensity map summed over all wavelengths equivalent to a regular image. Circles mark the reconstructed locations obtained as the average over all spectral peaks from one microcavity, and crosses mark the actual microcavity locations measured without the phantom. Scale bar, 50 μm. **e** Localization and size measurement of a large number (45) of microcavities below a phantom (1.1l^*^). Circle sizes are proportional to the reconstructed sizes of the microcavities. (Inset) Area marked by the red square imaged without the phantom. Scale bar, 500 μm. **f** The depth calibration curve obtained by measurement of the light distribution width of the transmitted signal at the phantom surface as a function of the microcavity depth. Each data point is the mean of the distribution width measured on 4 microcavities. For each microcavity the width was measured in four different directions. The error bars represent the standard error of the mean. The simulated curve was obtained by Monte Carlo simulation. (Insets) Images of the corresponding light distributions. Scale bar, 100 μm. **g** 3D reconstruction of the locations for the microcavities dispersed inside the phantom. The color of the spheres indicates the microcavity size and the image at the top shows the fluorescence intensity distribution at the phantom surface.

In general, if multiple light sources inside a scattering medium are too close together, their positions cannot be distinguished. In our case, this limitation was overcome by exploiting the unique emission spectra of each microcavity. For this purpose, a high-resolution spectrum was captured for each pixel of the image (a.k.a. a hyperspectral cube) by moving the spectrometer slit across the region of interest (Fig. 2a). Typically, a full hyperspectral scan took a few minutes, but a 10 s scan did not significantly affect the results (Supplementary Fig. 2). By displaying the fluorescence image at a particular wavelength, which matches the spectral peak of one microcavity, only that microcavity contributes to the image intensity (Fig. 2b). Its position can be determined by fitting a 2D Gaussian to this intensity map. Our method breaks the so-called optical diffusion limit by applying the same principle as photo-activated localization microscopy (PALM) and related techniques break the diffraction limit, i.e. by separating and analyzing the contributions from individual emission sources. However, while PALM relies on temporal separation of the signals, we separate them in the spectral domain (Supplementary Video 1). We name this new imaging method Diffuse Spectral Localization Imaging (DSLI). A single spectral peak was sufficient to localize the source (Fig. 2c), but having multiple peaks available from each source, typically 15–20, improved the accuracy and robustness of this procedure by a factor of 2.5 (Supplementary Figs. 3 and 4). The final reconstructed positions matched very well with the actual positions of the microcavities imaged without the phantom (Fig. 2d). Meanwhile, it is evidently not possible to determine their individual positions from the regular, spectrally unresolved image. The reconstructed positions at 1.9l^*^ were on average within 5 μm of their actual positions (determined by standard imaging with the phantom removed). The sizes and positions of a large number of microcavities could be retrieved with high accuracy and reliability (Fig. 2e). About 85 % of all the microcavities were successfully localized. The remaining microcavities could not be localized, mostly because they did not feature sharp spectral peaks. This could be attributed to contamination, irregular shape, or too small size of some microcavities. If we count only the microcavities with good spectra, 98% were successfully localized. No false positives were observed in any of the experiments.

Apart from the in-plane localization, the depth of the microcavities can also be determined. The width of the light distribution at the surface is directly related to the subsurface depth of the microcavity. Microcavities deeper within the scattering medium produce a wider light distribution. A depth calibration curve was obtained by simulation or by imaging microcavities at known depths and measuring the width of the light distribution at the surface (Fig. 2f). The calibration curve enables measurement of the microcavity depth with an accuracy of 40 μm and therefore three-dimensional localization of the microcavities embedded inside the scattering medium at a depth of 2.3 l^*^ (Fig. 2g). The depth localization accuracy was estimated by calculating the depths of multiple microcavities beneath a phantom of uniform thickness and taking the standard deviation. When the microcavities were embedded inside the scattering medium (Fig. 2g), the light distribution at the surface was slightly wider (~25%) compared with the case when the scattering medium was located only between the microcavities and the microscope objective. This difference has been taken into account by simulating both cases (Supplementary Fig. 5).

The unmixing algorithm is in principle not needed for spatial localization of the microcavities. However, if two or more microcavities were so close together that they could not be spatially separated, then the unmixing algorithm was used to identify which spectral peaks correspond to each microcavity, and only then the localization algorithm was performed at those specific peak wavelengths. The unmixing algorithm was also used after the localization to reconstruct the spectrum of the localized microcavities, especially if some of the peaks were missing, in order to calculate their sizes and external refractive indices.

### Microcavities inside cells for localization, tracking and sensing

**Fig. 3:**
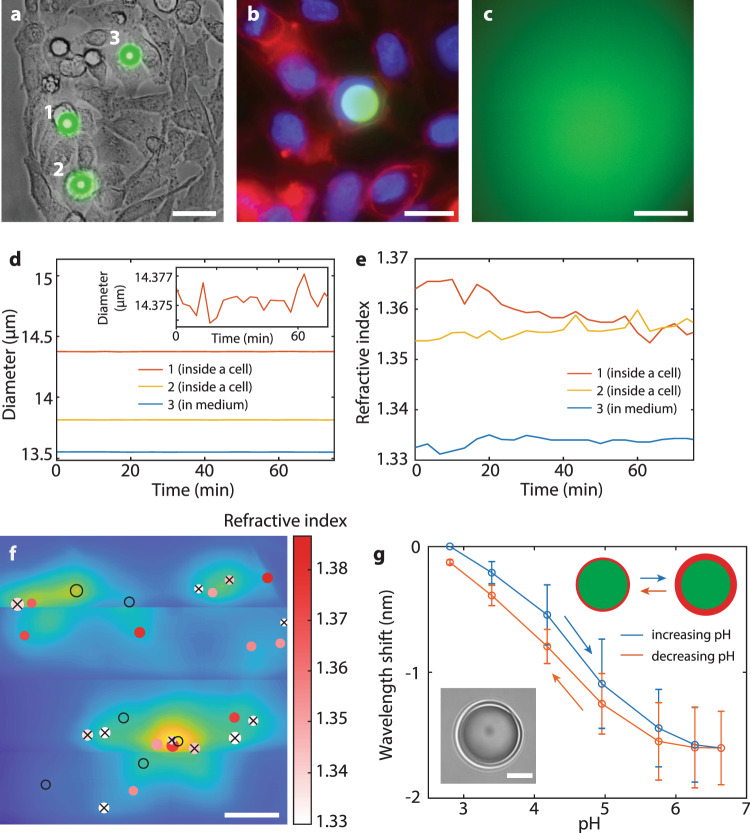
Simultaneous sensing and imaging. **a** Combined bright-field and fluorescence image of three microcavities, two of which are inside the cells (1 and 2). Scale bar, 30 μm. **b** Fluorescence image of a number of cells, one of them containing an internalized microcavity (green). The cell nuclei and cell membranes are stained in blue and red, respectively. Scale bar, 20 μm. **c** Fluorescence image of the area in **a** through a phantom (1.5l^*^). Scale bar, 100 μm. **d** The assessed diameters of three microcavities over time. (Inset) The assessed diameter of microcavity 1. **e** The assessed refractive indices in the surroundings of the same three microcavities over time. **f** Positions, sizes (proportional to the circle size) and surrounding refractive indices of a number of microcavities in a cell culture obtained with DSLI through a phantom (1.0l^*^). For the microcavities denoted by black empty circles, only their size was determined. Microcavities denoted by a black cross were identified as being outside the cells. The background image represents the fluorescent intensity distribution at the phantom surface. Scale bar, 500 μm. **g** Shift of the spectral peaks of microcavities coated with a pH-sensitive hydrogel was measured through a phantom (1.0l^*^) as the pH was changing. The plot represents the average response from 5 different microcavities, where the error bars represent the standard deviation. (Inset) Bright-field image of a coated microcavity. Scale bar, 5 μm.

**Fig. 4:**
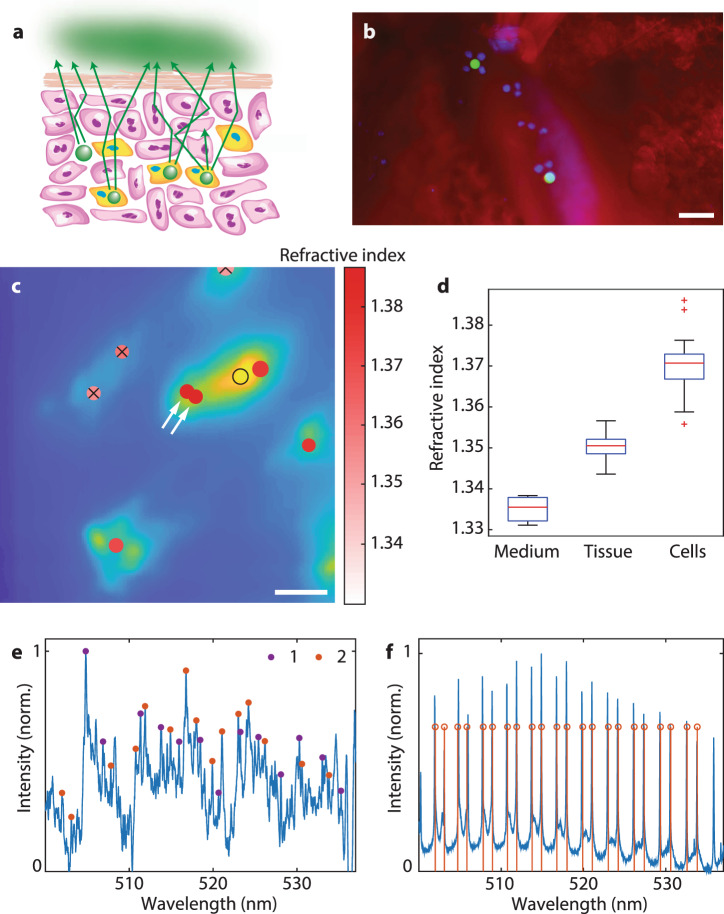
Sensing and imaging in biological tissues. **a** Schematics of the experiment. Some cells injected into the skin tissue (yellow) contained the microcavities. There were also free microcavities in the interstitial space. **b** Fluorescence image of a cross-section of skin tissue containing the injected microcavities. Only the injected cells had their nuclei stained with a blue dye, while the cell membranes of the tissue cells were stained with a red dye. Scale bar, 50 μm. **c** Reconstruction of microcavities injected below mouse skin represented in the same way as in Fig. 3f. The arrows denote two microcavities most likely located inside the same cell. The microcavity at the bottom of the image produced two intensity maxima due to tissue inhomogeneity, but the DSLI algorithm correctly identified this case as a single microcavity. Scale bar, 50 μm. **d** The assessed refractive indices of the microcavity surroundings in three different environments: in the cell growth medium (*n* = 20), injected below the skin of a mouse (*n* = 15), or uptaken by the cells (*n* = 25). The centerline, box limits, whiskers, and points indicate the median, upper and lower quartiles, extreme data points, and outliers, respectively. **e** Spectrum from two microcavities positioned below a 650 μm thick slice of brain tissue. **f** The reconstructed peak wavelengths (red) and the emission spectrum of one microcavity measured without the brain slice (blue).

To test the DSLI in real biological samples, the microcavities were introduced to HeLa cells, which readily internalized them by phagocytosis^34^ (Fig. 3a, b). The cells were in large excess compared to the microcavities. Typically the number of cells was 10 times larger than the number of microcavities, which led to 80–90% of the microcavities being internalized by the cells. On the other hand by having the microcavities in large excess up to 80–85% cells can contain at least one microcavity^35^. The cells were continuously imaged using DSLI through a phantom (Fig. 3c) for 80 min. The microcavity sizes and the surrounding refractive indices were calculated from the reconstructed spectra to a very high precision. The assessed size of each microcavity did not change over time (Fig. 3d) within the standard deviation of 0.6 nm (relative deviation of 4 × 10^−5^). Such accurate and robust determination of the microcavity sizes can be used for cell tagging, enabling tracking of thousands of cells for extended periods of time^20,35^. Since the refractive index inside the cells (1.37) is significantly higher than that of the cell growth medium (1.34) (Fig. 4d), we could also reliably determine whether a certain microcavity was located inside or outside a cell. From the measured refractive indices (Fig. 3e), it is clear that two microcavities were located inside the cell cytoplasm, while the third was in the growing medium, outside of the cell. The relative standard deviation of the refractive index measurements was 8 × 10^−5^. The refractive index of the cell medium was mostly constant within 0.002 RIU, while the cytoplasm refractive index was changing with time as much as 0.012 RIU, which could be attributed to different cellular processes. Similar behavior was obtained for different samples of cells (Supplementary Fig. 6). The ability to measure the refractive index enables robust identification of microcavities inside the cells in a larger sample (Fig. 3f) and precise long-term monitoring of the cell dynamics^25,26^. For 15% of the microcavities, because an insufficient number of peaks was identified due to low quality of their spectra, the assessment of the refractive index was not possible. However, the free spectral range value alone could still be used to determine the size, albeit at a lower accuracy of 6 nm.

### Functionalized microcavities for specific sensing

In order to measure tissue parameters beside its refractive index, the microcavities were coated with functional materials, whose optical properties change due to the external factor of interest. As a proof of concept, pH and temperature were measured. A 400–800 nm thick layer of pH-responsive polymer poly(methacrylic acid) (pMAA) or temperature-responsive polymer poly(N-vinylcaprolactam) (pNVCL), which are nontoxic and biocompatible, was coated onto the microcavities by means of initiated chemical vapor deposition (iCVD) technique^36–38^. iCVD enables the deposition of conformal coatings with nanometer thickness control in a solvent-free environment. The change in pH or temperature alters the swelling of the polymer, thereby also changing its refractive index. For the microcavities coated with the pH-responsive polymer, increasing the pH from 2.8 to 6.6 on average induced a large WGM shift of −1.6 nm (Fig. 3g), while the shift in the non-coated microcavities was negligible (+0.015 nm). The shift was reversible and repeatable. Taking into account the variations in the response between different microcavities and the small hysteresis, the resulting measurement accuracy was 0.4 pH units. The pH sensitive microcavities could be employed inside small tumors. It is known that solid tumors are characterized by a low pH, which induces spread of the tumor cells^39^, therefore the measurement of pH is very important in this case. Microcavities coated with the temperature-responsive polymer had a sensitivity of 44 pm/^∘^C at 37 ^∘^C (Supplementary Fig. 7).

### Localization of cells and sensing within tissues

A suspension of microcavities and cells containing the microcavities, so that typically, 75% of the microcavities were within cells, was injected with a hypodermic needle just below the skin of a mouse (Fig. 4a, b). The skin was later removed from the mouse to measure its thickness and optical properties. At *d* = 250–300 μm the skin thickness corresponds to ~ 1.7l^*^ at 532 nm. DSLI performed on the injected microcavities (Fig. 4c) resulted in similar size and refractive index accuracy as demonstrated using the phantoms. Due to tissue inhomogeneity, both lateral and depth accuracies were slightly worse compared to the phantoms, but nevertheless still sufficient to localize individual cells. The accuracies for a 300 μm thick skin were 9 μm in the lateral direction and 32 μm in depth. The lateral accuracy was determined in the same way as in the phantoms, that is by covering the microcavities with mouse skin and comparing the reconstructed locations with the actual positions determined without the skin. The depth accuracy was determined as the variation in the reconstructed depth of multiple microcavities located under the same piece of skin, so approximately at the same depth. To reconstruct the depth, a calibration curve was calculated by taking the average scattering coefficient of skin. Using a multi-layer model of skin to account for the different optical properties of its constituent tissues could help improve the accuracy of depth assessment.

Based on the measured refractive index, we could also identify which microcavities were within cells, and even whether more microcavities were inside the same cell. The refractive indices measured for microcavities in the cell growth medium, microcavities injected into tissue (but not inside the cells), and microcavities inside cells, showed statistically significant mutual differences in the refractive index (*p* < 10^−10^, two-sample *t*-test) (Fig. 4d). This enabled us to identify which microcavities are inside the cells even within tissue (Fig. 4c). Further, for any pair of microcavities which happened to be located within the same cell, the measured refractive index difference between the two was typically below Δ*n* = 0.003 (Supplementary Fig. 8). The two microcavities in Fig. 4c denoted by the arrows gave refractive indices of 1.3859 and 1.3865. Based on the refractive index distribution within cells (Fig. 4d), the probability that two beads inside separate cells have so close refractive index is <5%. Therefore, the probability that these two beads were within the same cell is >95 %. The fact that the two microcavities were also located very close to each other increases this probability even further. In real life applications of cell tracking it is important to know whether a microcavity is inside a cell, since we want to track cells, not the microcavities alone. It is also important to know if we are tracking two closely located cells or two microcavities in the same cell. The refractive index measurement enables such distinctions.

Microcavity probes were further tested in mouse brain tissue, either by using a layer of tissue with known thickness (Fig. 4e, f) or by injecting the microcavities directly into the brain with a hypodermic needle (Supplementary Fig. 9). The positions and sizes of the microcavities and refractive indices were successfully retrieved inside the brain up to a depth of 650 μm. For a 200 μm thick brain tissue, the lateral accuracy was 11 μm while depth accuracy was 25 μm. Biological results were reproducible. For both skin and brain, several tissue samples from different locations of the same animal as well as from different animals produced very similar results (Supplementary Fig. 10). While inhomogeneity, which is present with actual biological tissues, produces local discrepancies in the optical properties, these are not as important as the average sample characteristics (especially for thick and diffuse samples where discrepancies average out). This means that the diffuse image produced at the surface of the tissue, which is directly reproduced into spatial positions of microcavities by our method, is very similar for different tissue samples, as long as they are similar in terms of thickness and average optical properties (i.e. scattering coefficient and anisotropy).

### Longer emission wavelengths for deeper penetration

Here, as a proof of concept, microcavities emitting in green light (~520 nm) were employed. However, one obvious direction for the future would be to use longer wavelengths in order to decrease the scattering and therefore increase the penetration depth. For example, the demonstrated detection of the signal and identification of the microcavities at the depth of 3.5 l^*^ would correspond to ~4.5 mm in the mammalian cerebral cortex at a wavelength of 1100 nm^1,40^. To imitate light propagation at longer wavelengths, we used thicker phantoms with a smaller scattering coefficient. The lateral localization accuracy of DSLI when using a phantom with scattering coefficient comparable to that of skin at a wavelength of 730 nm and thickness of 360 μm (i.e., *d* = 1.5 l^*^) was 7 μm. This is slightly worse than for samples with a higher scattering coefficient due to the fact that the light distribution of the transmitted signal at the phantom surface is wider at a larger physical depth (Supplementary Fig. 11).

## Discussion

In conclusion, we have demonstrated deep tissue localization, tracking and sensing by using simple dye-doped polymer microcavities and commonly available optical equipment. In the future, other microcavities and microlasers with smaller size and longer wavelengths, such as semiconductor microcavities^20,41^, upconverting microlasers^42^ and spasers^43^ could also be employed. This could enable less invasive imaging, potentially even deeper in the tissue, and possibly even imaging of subcellular structures by labeling them with nanocavities.

DSLI in the present form in the terms of emission wavelength and particle size, could be already applied to a number of biological applications. A practical example where our method could complement other imaging modalities such as two-photon confocal, would be with the tumor models where tumorigenic cells are injected under the skin of a mouse. DSLI enables cell tracking over longer periods of time and simultaneous measurement of key chemical parameters (e.g. pH). Tracking of stem cells after their transplantation is another outstanding problem^44^, which could be tackled by the use of DSLI. Microcavities also enable physical sensing such as for example measurement of forces^26,45^ and surface tension^46^. When a fluid or soft microcavity is deformed by a force, its spectrum changes. While there are many force sensing methods, including force sensitive fluorescent molecules, most of these have not been applied to scattering tissues. For example, forces could be measured in the early tumorigenesis by the use of soft microcavities.

The microcavities could also be operated above the lasing threshold, which has its own advantages and disadvantages. The number of spectral peaks in the lasing regime is significantly lower. By using several gain materials emitting at different wavelengths, this could enable simultaneous identification of multiple microcavities from a single spectrum^20^. On the other hand, the smaller number of spectral peaks makes calculation of the microcavity size and refractive index less accurate (and impossible in the case of single-mode emission). Moreover, achieving the lasing regime deep inside the tissue may require high excitation power, which might damage the tissue. However, this could be mitigated by the use of semiconductor lasers with low lasing thresholds. By making the microcavity output sensitive to calcium, neural activity could also be measured deep inside the brain. Additionally, microcavities could be combined with other imaging modalities, such as multiphoton excitation, or as a guidestar to further increase the imaging depth and resolution of various imaging techniques. Due to its relative simplicity and multimodality, the DSLI could thus become a powerful method for imaging deep inside biological tissues.

## Methods

### Microcavities

Green fluorescent polystyrene microspheres (Thermo Scientific, Fluoro-Max), with mean diameter of 15 μm, coefficient of variation of 14%, refractive index of 1.59 at 589 nm, and absorption/emission maxima at 468 nm/508 nm, were used as WGM microcavities. The microspheres were mixed into Cytop CTL 109A polymer with the refractive index similar to that of water (~1.34). The polymer was drop-coated to a glass slide and the solvent was left to evaporate for 24 hours at room temperature to form a thin (~30 μm) layer. When microspheres were supplied to the cell culture, the beads were first dispersed in PBS at 1.2 mg/ml and sterilized for 15 min at 100 ^∘^C. The dispersion was sonicated in a water bath sonicator before adding to the cell culture.

### Phantoms

The tissue phantom layers^47^ were made of polystyrene microspheres (0.51 μm diameter, 5–10% coefficient of variation) dispersed in polydimethylsiloxane (PDMS) polymer (SYLGARD 184). The PDMS was polymerized between two glass plates to form a free-standing layer of predefined thickness (20–500 μm), which was inserted between the layer containing the microcavities and the microscope objective. Unless stated otherwise, the phantoms had the following scattering properties at 532 nm: *μ*_*s*_ = 63 mm^−1^, *g* = 0.87 and l^*^ = 120 μm, similar to that of strongly scattering biological tissues, like human epidermis. These parameters were calculated from the size, refractive index, and concentration of the scatterers using Mie theory^48^. The optical properties of the phantoms were verified with measurements of diffuse reflectance and total transmittance at a minimum of 5 different positions across each sample using an integrating sphere. The sample was placed on one port of the sphere (Supplementary Fig. 12) and the other ports were closed. The light source was a green laser (532 nm, 5 mW), similar to the emission wavelength of the microcavities. For measuring the total transmittance, the sample was placed on the front port of the sphere. For measuring diffuse reflectance the sample was placed on the back port. For a particular phantom thickness, Monte Carlo simulation was performed using the scattering properties of the phantom material, including the phase function, calculated from Mie theory. The resulting values of transmittance and reflectance were compared with the measured ones (Supplementary Figs. 13 and 14). In simulations an absorption coefficient of *μ*_*a*_ = 0.2 mm^−1^ was used. Because *μ*_*a*_ is small compared to *μ*_*s*_ it had a very modest impact on the simulation results. Varying *μ*_*a*_ from 0 to 2 mm^−1^ changed the simulation results by less than 5 %. For the mouse skin, where the optical properties were not known in advance, the same procedure was performed in reverse to assess the scattering parameters from measured reflectance and total transmittance. Specifically, Monte Carlo simulation was performed while varying both *μ*_*s*_ and *g* in a meaningful range until the simulated transmittance and reflectance matched the measured ones.

### Optical setup and spectral data analysis

Excitation of the microcavities and collection of light was performed in an epifluorescence configuration through a 10×, 0.3 NA or a 20×, 0.45 NA objective. The excitation source was a blue LED (450 nm) with a power of 100–200 mW at the sample. Collected light was sent into an imaging spectrometer with 10–40 μm wide slit and a CCD detector with a resolution of 1600 × 200 pixels. Andor Solis software was used to collect the spectra. Typically, 100–200 line scans, with 0.1–5 s exposure time each were performed, resulting in the scanning times of 10–500 s for the full hyperspectral scan. For the slower scans, a typical hyperspectral image had 200 × 200 × 1600 points (the third number being the spectral dimension) and covered a physical size on the sample of 320 μm × 320 μm for 10× objective. The spectral resolution was 0.023 nm. For faster scans we used binning, so that the final image was 100 × 100 × 800, covering the same physical size and same spectral range. To image larger sample areas, multiple scans were stitched together. Raw data was transformed to a 200 × 200 × 1600 matrix in MATLAB. The spectrum in each pixel of the image was corrected by subtracting the fluorescent background, that is a smooth curve corresponding to the emission of the fluorescent dye alone, which is the same for all microcavities. The spectral sum was obtained by summing the corrected spectra together. The peaks are the local maxima with the prominence higher than the defined threshold.

### Whispering gallery modes

Experimentally measured spectral peaks were fitted to the exact solution for a spherical resonator^32,33^. Fulfilling the boundary conditions for the eigenmodes of electromagnetic field in a spherical resonator results in the characteristic equation for the eigenfrequencies for transverse electric (TE) and transverse magnetic (TM) modes. A similar approach was used to calculate the eigenfrequencies in a multilayer spherically symmetric microcavity^49^ for microcavities coated with a hydrogel layer to determine the required layer thickness. The general solutions in each layer are the spherical Bessel and Hankel functions, the same as for non-coated spheres. Each additional layer produces another set of equations that stitch the solutions on each interface accordingly. Satisfying all boundary conditions simultaneously results in the characteristic equation for the eigenfrequencies. This equation was solved numerically to get the eigenfrequencies of the WGMs. The spectral positions of WGMs in the case of a simple non-coated sphere are determined by three parameters: diameter of the spherical microcavity, internal refractive index, and external refractive index. Any two of the three parameters can be measured by fitting the experimental data to the theoretical model, while the third parameter has to be known in advance. In our experiments, the internal refractive index is known, so the external refractive index and size are calculated by overlapping the experimental and theoretical peak positions for a range of possible diameters and external refractive indices.

### Unmixing algorithm

The central wavelength *λ*_*n*_ of each peak was retrieved by fitting the experimental emission spectrum to a Lorentzian curve. The peaks have to be grouped so that each group is associated with one microcavity. The grouping was based on the fact that the peaks are equally spaced in frequency and this spacing as well as the position of peaks is different for each microcavity size. If two peaks *i* and *j* belong to the same microcavity there should be other peaks, which are separated from these two peaks by an equal free spectral range (FSR) value defined as

1 $${{{\mbox{FSR}}}}_{i,j}=1/{\lambda }_{i}-1/{\lambda }_{j}.$$

To find *i* and *j* with this property, we propagated *FSR*_*i*,*j*_ value across the spectrum as

2 $$1/{\widetilde{\lambda }}_{i,j}^{N}=1/{\lambda }_{i}+N\cdot {{{\mbox{FSR}}}}_{i,j},$$

where *N* is an integer. The propagated ${\widetilde{\lambda }}_{i,j}^{N}$ values were compared to the wavelengths of the measured peaks *λ*_*n*_ (Supplementary Fig. 1). This calculation was repeated for every peak *j* and the one that produced the best overlapping between measured and propagated peak wavelengths gave the correct FSR value of peak *i*. This algorithm was then repeated for every peak *i*, so we ended up with all of the peaks having been assigned their respective FSR values. Grouping was then achieved by either comparing the FSR values or by propagating them across the spectrum and grouping the peaks that overlap. In the latter example, two groups of peaks are found for each microcavity, one for TE and the other for TM polarization. Since both polarizations have the same FSR value, they can be associated with a single microcavity. The last step of the algorithm was to fill in the positions of potentially missing peaks, which was performed by simply taking into account the FSR value.

### Cell and tissue samples

HeLa cells were seeded onto 35 mm dish (Ibidi, μ-dish) and incubated overnight in the complete culturing medium (DMEM medium with 10% fetal bovine serum and 1% pen-strep) at 5% CO_2_ and 37 ^∘^C to be 30% confluent. The microcavities dispersed in PBS were added to the cell culture medium to a final concentration of 20 μg/ml and incubated for 48 h, by which the cells reached 80% confluency. When imaging internalization of the microcavities into HeLa cells, the cell membrane was labeled with fluorescent dye CellMaskOrange (Molecular Probes) and the nucleus with Hoechst 33342 (Thermo Fisher Scientific). Freshly sacrificed female albino mice, FVB/N, ~55 weeks old were used for tissue experiments. A dispersion of microcavities in PBS or a dispersion of cells containing the microcavities was injected just below the skin of the mouse hind right leg. For regular fluorescent imaging, the mouse skin tissue was labeled with fluorescent dye CellMask Orange and the injected HeLa cells were labeled with Hoechst 33342. To get brain tissue, the mouse head was decapitated, the skin from the upper part of the head was removed, followed by the removal of the skull with a scalpel and tweezers. The skin or brain was placed in a sterile PBS and later transferred to the objective glass. The scattering coefficient of skin was measured with an integrating sphere in the same way as described for phantoms and agrees with typically reported values^1,50^.

### Functionalized microcavities

The iCVD deposition was run simultaneously on the microcavities dispersed as a monolayer in a Petri dish and for reference on a piece of Si wafer. A custom build reactor setup was used as described in ref. ^51^. The monomers methacrylic acid (MAA) and di(ethylene glycol) divinyl ether (DEGDVE, 99%; Aldrich, Germany) were heated to 70 ^∘^C, while the N-vinylcaprolactam (NVCL, 98%; Aldrich, Germany) was heated to 78 ^∘^C and they were fed into the reactor through a heated mixing line. The flow rates were controlled with needle valves. The initiator tert-butylperoxide (TBPO) was kept at room temperature and fed into the reactor through a mass flow controller. The flow rates were 0.25 ± 0.05 sccm for NVCL, 0.35 ± 0.15 sccm for DEGDVE and 1.02 ± 0.05 sccm for TBPO for the pNVCL deposition. For the pMAA deposition, the flow rates were 4 sccm for MAA, 0.4 sccm for DEGDVE and 1 sccm for TBPO. The depositions were performed at a constant working pressure of 350 mTorr. The substrate and filament temperatures were 35 ^∘^C and 250 ^∘^C, respectively. The polymer layers were grown up to a thickness of 400 nm for the pNVCL and to 800 nm for the pMAA. The thickness was monitored in situ with laser interferometry on the Si wafer. The thickness of the polymer thin films and the temperature-dependent swelling were evaluated on the silicon substrate, using spectroscopic ellipsometry (J.A. Woollam M-2000). Data acquisition was performed in air at three angles (65, 70, and 75^∘^) in the wavelength range from 370 to 1000 nm. Data were evaluated using simulations with an optical model comprised of three layers; the silicon substrate, the native silicon dioxide (1.7 nm) and a Cauchy layer representing the polymer. The temperature-induced swelling in water was also explored with in situ ellipsometry at a fixed angle of 75^∘^, utilizing a heated liquid cell (Woollam, USA). The temperature was ramped from 12 ^∘^C to 50 ^∘^C at a rate of 1 ^∘^C/min. The effective medium approximation (EMA) was used to model the composite consisting of polymer and water. The model mixes the optical constants of water with those of the dry Cauchy layer (i.e. polymer) according to their relative fraction (which is the fitting parameter). The pH-responsive microcavities were tested in citrate-phosphate buffer, which was prepared by mixing 20 mM disodium phosphate and 10 mM citric acid in an appropriate ratio to achieve the desired pH.

### Statistics and reproducibility

Unless stated otherwise, the error bars in the plots represent standard error of the mean. To make a statistical distinction between given data points a two-sample *t*-test is performed. Sample sizes are provided in the captions of each individual figure. Localization and spectral unmixing measurements on non-biological samples were repeated tens of times. Localization and spectral unmixing on biological tissues were performed on at least 15 samples collected from a total of 5 animals. The experiments with cells were reproduced for 8 independent cell cultures. To measure pH and temperature response, a total of 9 and 5 coated microcavities were used, respectively. The reproduced experiments led to the same conclusions within the measurement error. Reproducibility was ensured for all the representative microscope images by repeating the same acquisition for a range of at least 5 different positions on at least 3 distinct samples.

### Reporting summary

Further information on research design is available in the Nature Research Reporting Summary linked to this article.

## Supplementary information

Supplementary Information

Description of Additional Supplementary Files

Supplementary Video 1

Reporting Summary

## Data Availability

The data generated in this study have been deposited in the Zenodo database under accession code 10.5281/zenodo.5809094.

## Code availability

The code developed in this study has been deposited in the Zenodo database under accession code 10.5281/zenodo.5809094.

## References

[1] Jacques SL (2013). Optical properties of biological tissues: a review. Phys. Med. Biol..

[2] Ntziachristos V (2010). Going deeper than microscopy: the optical imaging frontier in biology. Nat. Methods.

[3] Theer P, Denk W (2006). On the fundamental imaging-depth limit in two-photon microscopy. J. Opt. Soc. Am..

[4] Tkaczyk, E. R. Innovations and developments in dermatologic non-invasive optical imaging and potential clinical applications. *Acta Derm-Venereol* 5 (2017). 10.2340/00015555-2717 PMC5943168 28676880

[5] Yoon, S. et al. Deep optical imaging within complex scattering media. *Nat. Rev. Phys*.1–18 (2020).

[6] Wang, L. V. *Photoacoustic imaging and spectroscopy* (CRC Press, 2017).

[7] Ruan H, Liu Y, Xu J, Huang Y, Yang C (2020). Fluorescence imaging through dynamic scattering media with speckle-encoded ultrasound-modulated light correlation. Nat. Photonics.

[8] Jiang, H. *Diffuse optical tomography: principles and applications* (CRC Press, 2018).

[9] Yu H (2015). Recent advances in wavefront shaping techniques for biomedical applications. Curr. Appl. Phys..

[10] Velasco MGM (2021). 3d super-resolution deep-tissue imaging in living mice. Optica.

[11] Maric D (2021). Whole-brain tissue mapping toolkit using large-scale highly multiplexed immunofluorescence imaging and deep neural networks. Nat. Commun..

[12] Ntziachristos V, Tung C-H, Bremer C, Weissleder R (2002). Fluorescence molecular tomography resolves protease activity in vivo. Nat. Med..

[13] Stuker F, Ripoll J, Rudin M (2011). Fluorescence molecular tomography: principles and potential for pharmaceutical research. Pharmaceutics.

[14] Ozturk MS (2021). Intravital mesoscopic fluorescence molecular tomography allows non-invasive in vivo monitoring and quantification of breast cancer growth dynamics. Commun. Biol..

[15] Yang F (2018). Improving mesoscopic fluorescence molecular tomography via preconditioning and regularization. Biomed. Opt. Express.

[16] Dang X (2019). Deep-tissue optical imaging of near cellular-sized features. Sci. Rep..

[17] Moretti C, Gigan S (2020). Readout of fluorescence functional signals through highly scattering tissue. Nat. Photonics.

[18] Welsher K (2009). A route to brightly fluorescent carbon nanotubes for near-infrared imaging in mice. Nat. Nanotechnol..

[19] Fan X, Yun S-H (2014). The potential of optofluidic biolasers. Nat. Methods.

[20] Martino N (2019). Wavelength-encoded laser particles for massively multiplexed cell tagging. Nat. Photonics.

[21] Schubert M (2015). Lasing within live cells containing intracellular optical microresonators for barcode-type cell tagging and tracking. Nano Lett..

[22] Toropov N, Vollmer F (2021). Whispering-gallery microlasers for cell tagging and barcoding: the prospects for in vivo biosensing. Light Sci. Appl.

[23] Tang S-J (2021). Laser particles with omnidirectional emission for cell tracking. Light Sci. Appl..

[24] Toropov N (2021). Review of biosensing with whispering-gallery mode lasers. Light Sci. Appl..

[25] Schubert M (2020). Monitoring contractility in cardiac tissue with cellular resolution using biointegrated microlasers. Nat. Photonics.

[26] Humar M, Yun SH (2015). Intracellular microlasers. Nat. Photonics.

[27] Cho S, Humar M, Martino N, Yun SH (2016). Laser particle stimulated emission microscopy. Phys. Rev. Lett..

[28] Fernandez-Rosas E (2009). Intracellular polysilicon barcodes for cell tracking. Small.

[29] Gratton SE (2008). The effect of particle design on cellular internalization pathways. Proc. Natl. Acad. Sci. U.S.A.

[30] Fernández-Rosas E (2010). Internalization and cytotoxicity analysis of silicon-based microparticles in macrophages and embryos. Biomed. Microdevices.

[31] Humar M, Dobravec A, Zhao X, Yun SH (2017). Biomaterial microlasers implantable in the cornea, skin, and blood. Optica.

[32] Matsko AB, Ilchenko VS (2006). Optical resonators with whispering gallery modes I: Basics. IEEE J. Sel. Top. Quant..

[33] Gorodetsky ML, Fomin AE (2006). Geometrical theory of whispering-gallery modes. IEEE J. Sel. Top. Quant..

[34] Schubert M (2017). Lasing in live mitotic and non-phagocytic cells by efficient delivery of microresonators. Sci. Rep..

[35] Humar M, Upadhya A, Yun SH (2017). Spectral reading of optical resonance-encoded cells in microfluidics. Lab. Chip..

[36] Coclite AM (2013). 25th anniversary article: CVD polymers: a new paradigm for surface modification and device fabrication. Adv. Mater..

[37] Muralter F, Perrotta A, Werzer O, Coclite AM (2019). Interlink between tunable material properties and thermoresponsiveness of cross-linked poly (N-vinylcaprolactam) thin films deposited by initiated chemical vapor deposition. Macromolecules.

[38] Ghasemi-Mobarakeh L (2019). Manipulating drug release from tridimensional porous substrates coated by initiated chemical vapor deposition. J. Appl. Polym. Sci..

[39] Thews O, Riemann A (2019). Tumor ph and metastasis: a malignant process beyond hypoxia. Cancer Metast. Rev..

[40] Horton NG (2013). In vivo three-photon microscopy of subcortical structures within an intact mouse brain. Nat. Photonics.

[41] Fikouras AH (2018). Non-obstructive intracellular nanolasers. Nat. Commun..

[42] Liu Y (2020). Controlled assembly of upconverting nanoparticles for low-threshold microlasers and their imaging in scattering media. ACS Nano.

[43] Galanzha EI (2017). Spaser as a biological probe. Nat. Commun..

[44] Bulte JW, Daldrup-Link HE (2018). Clinical tracking of cell transfer and cell transplantation: trials and tribulations. Radiology.

[45] Manzo M, Cavazos O, Ramirez-Cedillo E, Siller HR (2020). Embedded spherical microlasers for in vivo diagnostic biomechanical performances. J. Eng. Sci. Med. Diagn. Therapy.

[46] Pirnat G, Humar M (2021). Whispering gallery-mode microdroplet tensiometry. Adv. Photonics Res.

[47] de Bruin DMM (2010). Optical phantoms of varying geometry based on thin building blocks with controlled optical properties. J. Biomed. Opt..

[48] Prahl, S. et al. Mie scattering calculator (2007). https://omlc.org/calc/mie_calc.html.

[49] Cohoon D (1989). An exact solution of mie type for scattering by a multilayer anisotropic sphere. J. Electromagn. Wave..

[50] Taddeucci A, Martelli F, Barilli M, Ferrari M, Zaccanti G (1996). Optical properties of brain tissue. J. Biomed. Opt..

[51] Ranacher C (2015). Layered nanostructures in proton conductive polymers obtained by initiated chemical vapor deposition. Macromolecules.

