# Supplementary material for: Deep tissue localization and sensing using optical microcavity probes
Source: Nat Commun. 2022 Mar 11;13:1269. doi: 10.1038/s41467-022-28904-6 (PMC8917156; doi:10.1038/s41467-022-28904-6)
Supplement: Supplementary file 1 — Supplementary Information [file 41467_2022_28904_MOESM1_ESM.pdf]

Supplementary Information

Deep tissue localization and sensing using optical microcavity probes

Aljaž Kavčič, Maja Garvas, Matevž Marinčič, Katrin Unger, Anna Maria Coclite, Boris Majaron, and Matjaž Humar

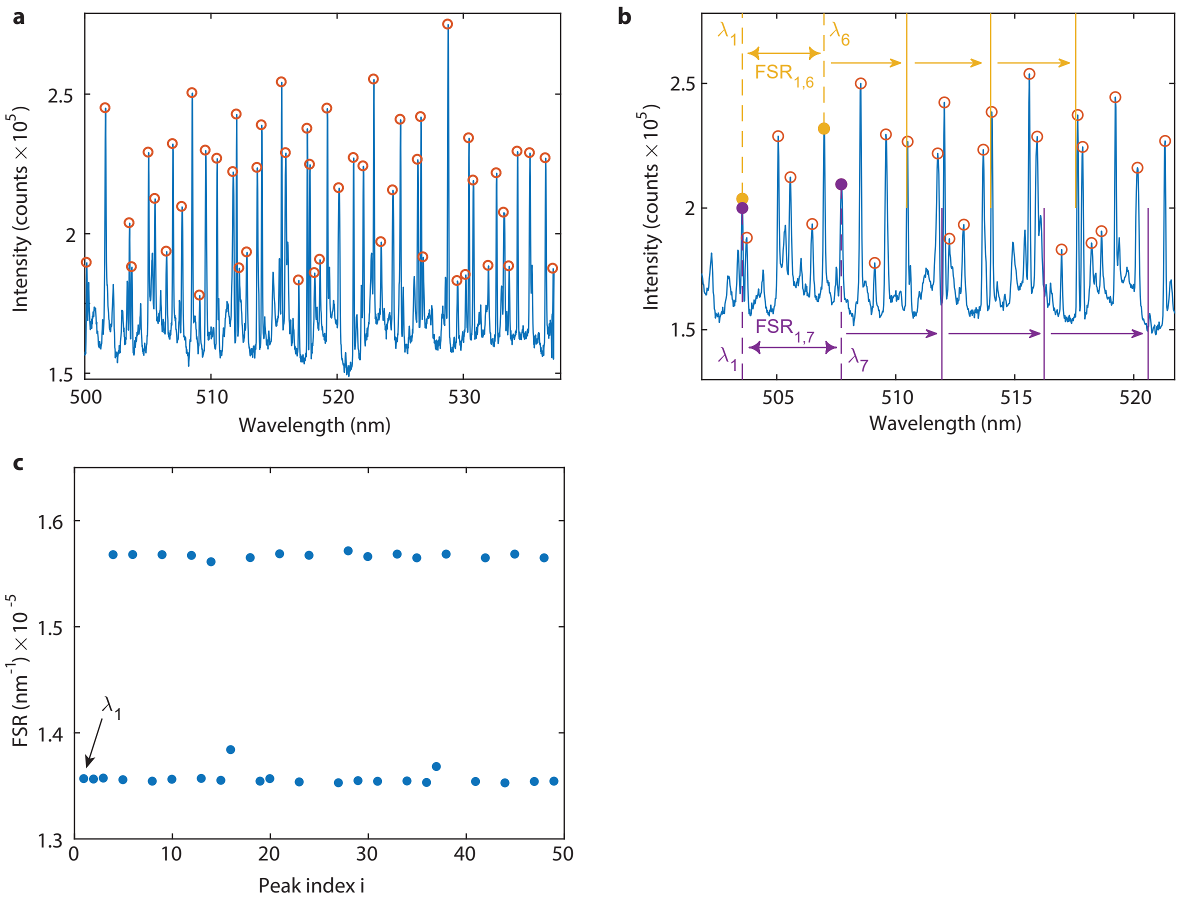

Supplementary Figure 1: Working principle of the unmixing algorithm. **a)** A combined emission spectrum from two microcavities collected through a phantom. From the spectrum alone it is not obvious which peaks correspond to which microcavity. **b)** Free spectral range (FSR) is calculated for two peaks and propagated to longer wavelengths. If peaks 1 and 6 are chosen, the propagated wavelengths closely match the peaks in the spectrum, meaning that these peaks correspond to a single microcavity. The corresponding $\mathrm{FSR}_{1,6}$ value is assigned to λ_1 and is plotted in **c**. Conversely, if peaks 1 and 7 are chosen, the propagated values do not match the peaks in the spectrum, therefore these two peaks are not associated to the same microcavity. **c)** When FSR values for all the peaks are plotted, they form two distinctive sets, each corresponding to one microcavity.

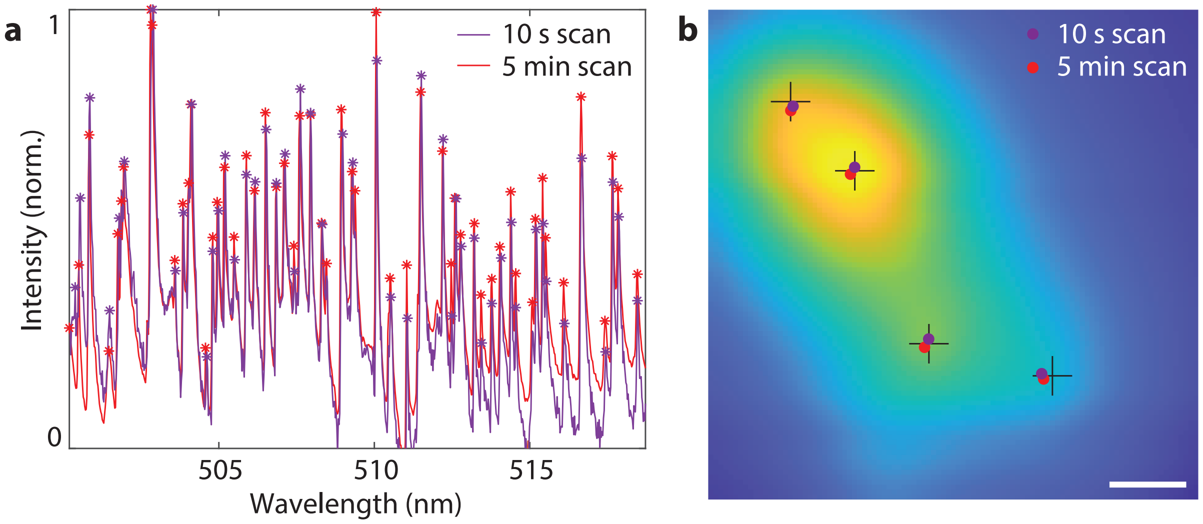

Supplementary Figure 2: The effect of the hyperspectral acquisition speed on the results. **a)** Spectra acquired from the region in **b** at two scanning speeds through a 1.0 l^* thick phantom layer. Both spectra are comparable once normalized and the same spectral peaks can be identified. **b)** Crosses mark the actual microcavity locations measured withot the phantom, while circles mark the reconstructed locations. For a 10 s scan and 5 min scan, the reconstructed localization accuracy was 4.2 µm and 5.0 µm, respectively. The reconstructed positions for both cases were on average 1.3 µm apart from each other. Overall, within the measurement error there are virtually no differences in the results are obtained by a 10 s or 5 min scan. Scale bar, 50 µm.

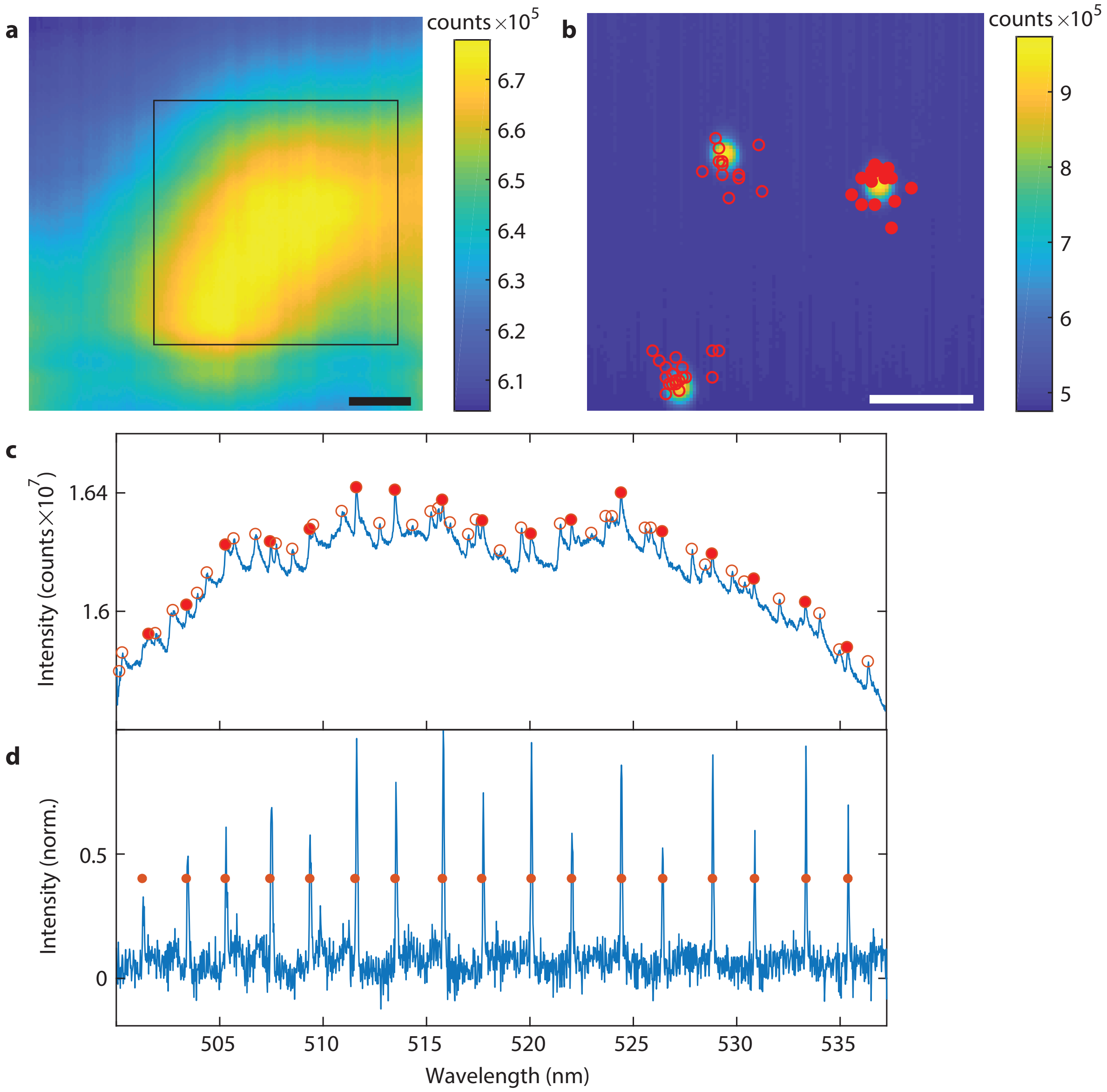

Supplementary Figure 3: Example of microcavity localization. **a)** Regular image through a 1.7 l^* phantom derived as a sum over all wavelengths in the hyperspectral image. **b)** Results of the localization algorithm (red circles) superimposed on the image of the microcavities acquired without the phantom for the region marked by the black square in **a**. Each point corresponds to the localization result performed on a single wavelength. Since each microcavity has multiple spectral peaks, multiple points are present. **c)** Cumulative spectrum from the entire region shown in **a**, before removal of the fluorescence background. The circles mark all identified peaks. Red dots correspond the right microcavity in **a**, while the empty circles correspond to the other two microcavities. **d)** Reconstructed peaks for one of the three microcavities (red) and its spectrum obtained without the phantom (blue). The corresponding locations are clustered in the group marked with full circles in **b**. Scale bars, 50 µm.

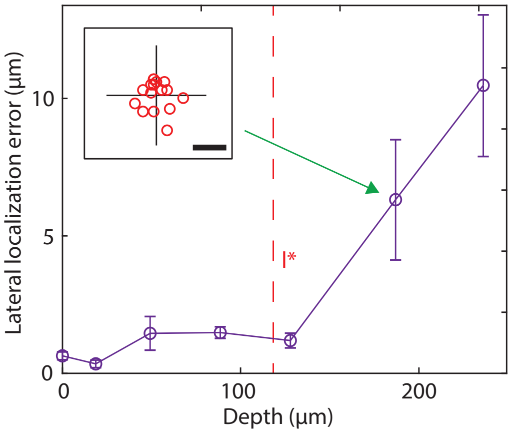

Supplementary Figure 4: Each spectral peak in the spectrum of one microcavity is used separately to reconstruct the microcavity location (red circles in the inset). The final location is calculated as the average over all the peaks. The resulting standard error of the mean location is plotted as a function of the phantom thickness. Below the transport length l^*, the error is nearly constant, but increases when transitioning into the diffusive light propagation regime. Four different microcavities were used to derive the statistics. Inset: Example of microcavity location determination derived from individual spectral peaks (red circles). The black cross in the inset indicates the actual microcavity location. Scale bar, 20 µm.

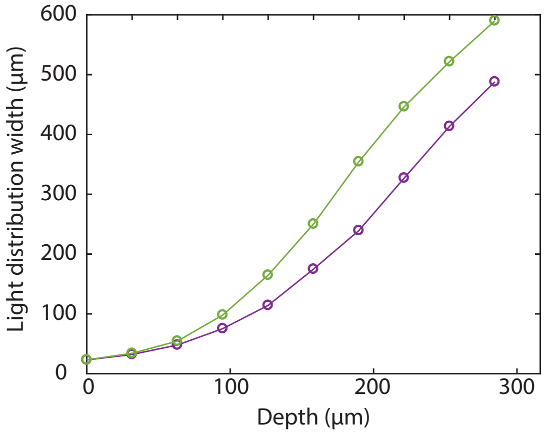

Supplementary Figure 5: Monte Carlo simulation of the light distribution width of the transmitted signal at the phantom surface as a function of the depth for two cases: microcavities completely embedded in the scattering medium (green) and with only a layer of the scattering medium inserted between the microcavities and the microscope objective (purple).

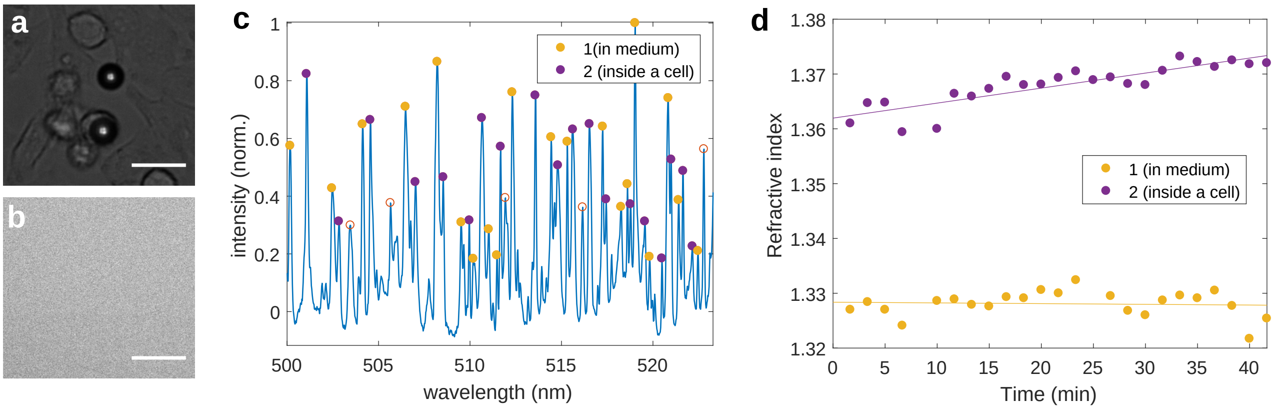

Supplementary Figure 6: An example of a time-dependent measurement of the refractive index from microcavities in a cell culture. (**a**) Brightfield image of two microcavities, bottom one inside a cell and the top one outside. (**b**) Fluorescence image of the same area covered with a scattering layer with its thickness corresponding to 1.5 l^*. Scale bar, 25 µm. **c)** The identified peaks in the cumulative spectrum that are grouped according to their reproduced value of free spectral range. **d)** The calculated values of the refractive indices from the reconstructed peak positions for the two microcavities. The refractive index of microcavity inside the cell is higher and is changing, while the refractive index of the microcavity in the cell medium remains constant.

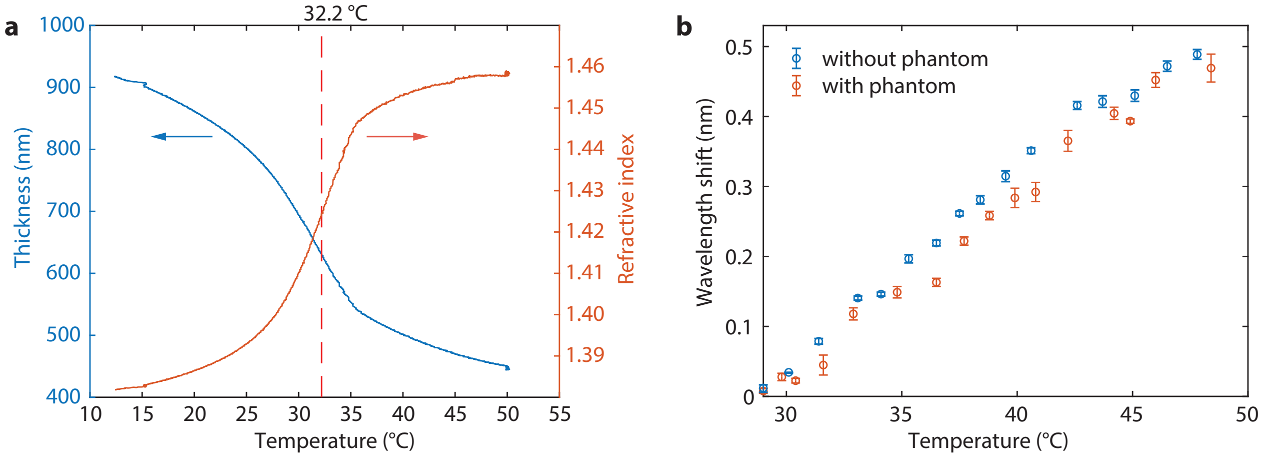

Supplementary Figure 7: Temperature-responsive hydrogel coating. **a)** Ellipsometric data of a thin film of responsive hydrogel deposited onto a silicon substrate. Shrinkage of the polymer thin film was observed with increasing temperature. As a result the refractive index increased with temperature. The vertical dashed line represents the lower critical solution temperature of 32.2 °C. **b)** Temperature shift of 6 WGM peaks of a single microcavity coated with the temperature-responsive hydrogel. With the temperature change from 30 to 48 °C, the thickness of the hydrogel coating decreased from 693 to 450 nm and the refractive index increased from 1.4103 to 1.4587, resulting in a WGM shift of +0.5 nm. The average sensitivity of 5 microcavities was 44 pm/°C with a standard deviation of 23 pm/°C. The different sensitivity of each microcavity is probably caused by slight variations in the coating thickness. For uncoated microcavities, the WGM shift was −0.17 nm, due to the slight changes of the refractive indices both inside and outside of the microcavity, as well as its size. The results obtained with and without an overlaid phantom (1.5 l^*) agree very well.

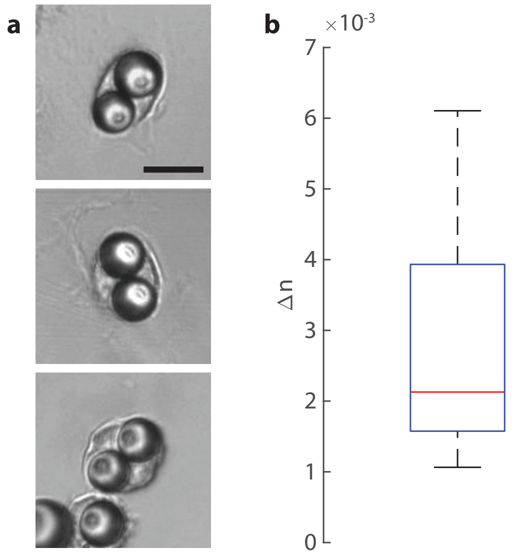

Supplementary Figure 8: Multiple microcavities inside the same cell. **a)** Three examples of pairs of microcavities within the same cell. Scale bar, 20 µm. **b)** Distribution of the measured refractive index difference between 17 pairs of microcavities within the same cell. The centerline, box limits and whiskers indicate the median, upper and lower quartiles and extreme data points, respectively.

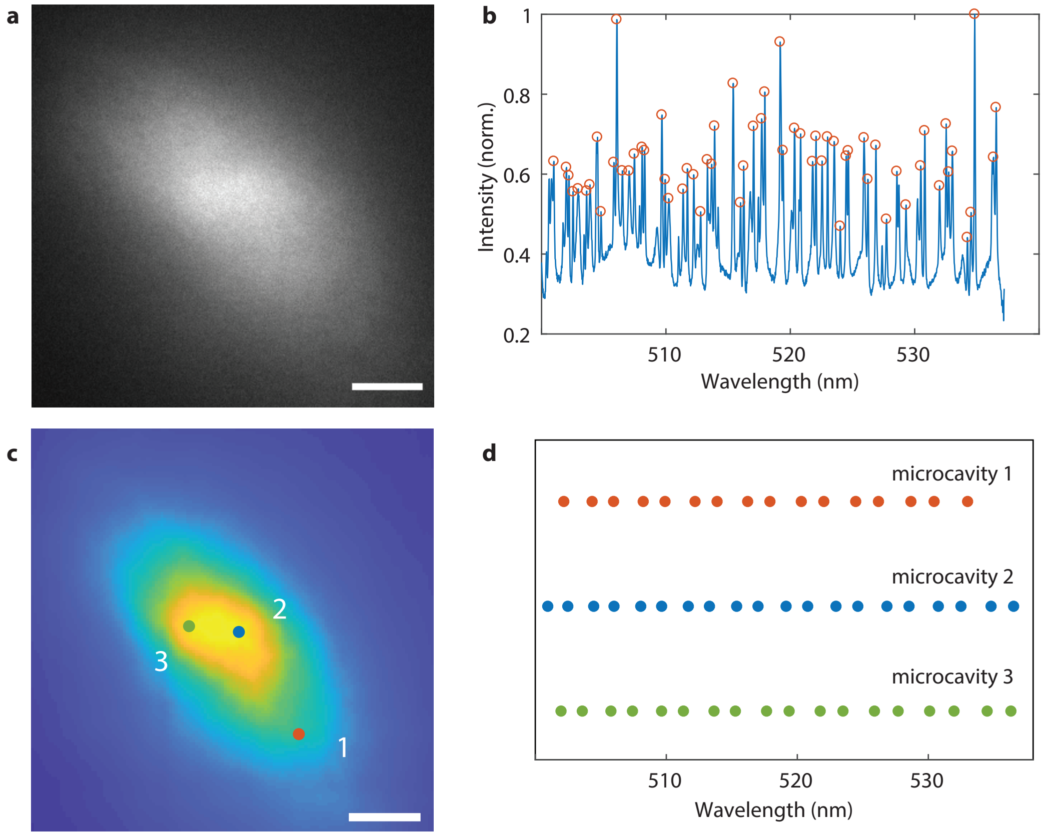

Supplementary Figure 9: Microcavities injected into mice brain. **a)** Fluorescence image taken with a normal camera. **b)** Spectrum from the same area with the identified peaks. **c)** Three microcavities localized within the tissue and **d)** the corresponding reconstructed peak wavelengths of the three microcavities. Scale bars, 20 µm.

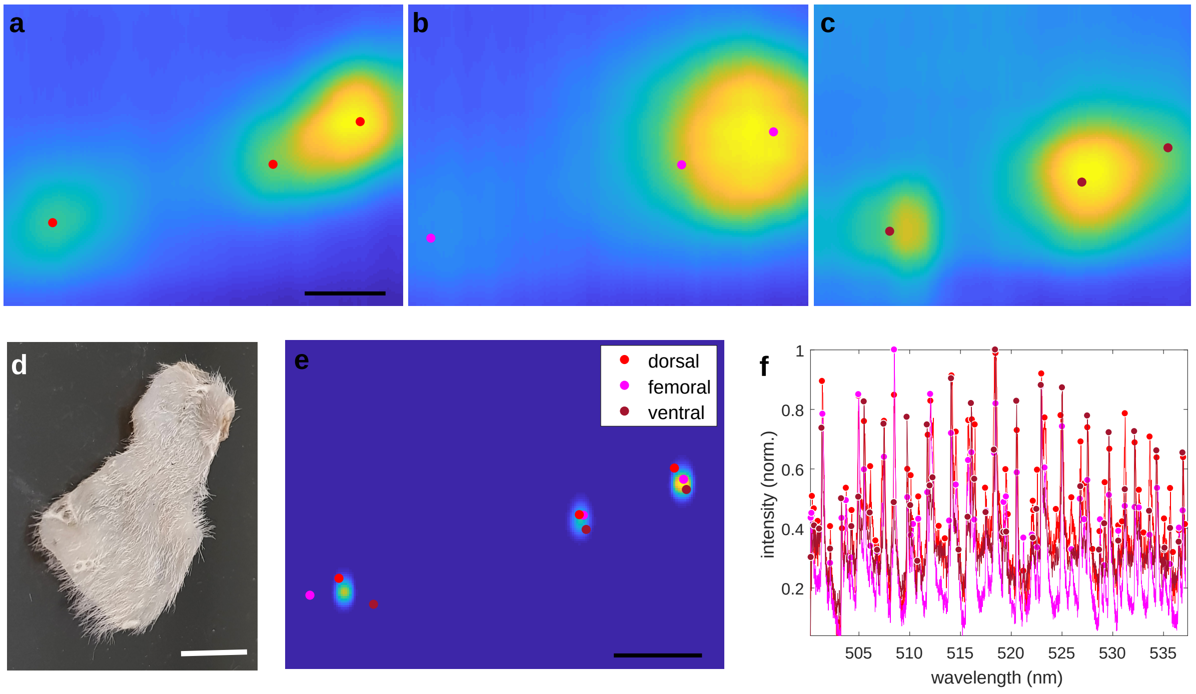

Supplementary Figure 10: Localization of the same three microcavities embedded in a polymer layer and covered with skin samples collected from different body parts of a mouse, specifically from the **a)** dorsal, **b)** femoral (hind leg) and **c)** ventral locations. Scale bar, 25 µm. **d)** Mice skin sample from the femoral location of a hind leg. Scale bar, 5 mm. **e)** Comparison of the localization results superimposed onto the image of the microcavities captured without the skin sample. The average localization accuracy was 8.7 µm, 10.7 µm and 9.3 µm for the dorsal, femoral and ventral side respectively. Scale bar, 25 µm. **f)** Normalized spectra from **a**, **b** and **c**. The identified peaks and their positions are almost the same.

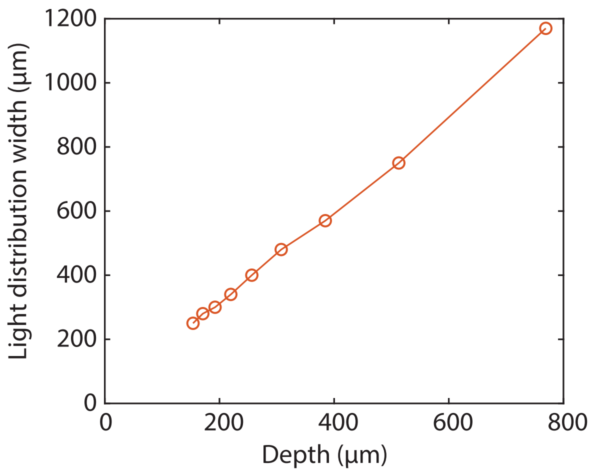

Supplementary Figure 11: Simulated light distribution width of the transmitted signal at the phantom surface as the function of a microcavity depth, where the optical thickness is kept constant at 2 l^*. To achieve a constant optical thickness, the scattering coefficient is changed as $\mu_s = 2/(D * (1 - g))$, where D is depth and $g = 0.87$. As an example, for a microcavity 100 µm deep a scattering coefficient of 150 mm^{-1} is used and for a microcavity twice as deep at 200 µm a scattering coefficient of 77 mm^{-1} is used.

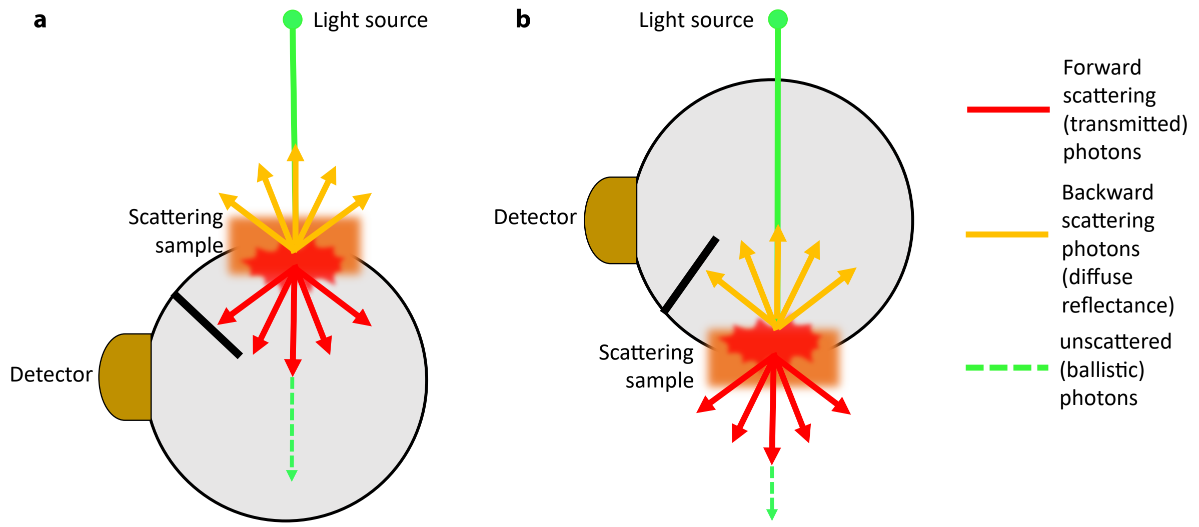

Supplementary Figure 12: Measurement of scattering properties of phantoms and biological samples using an integrating sphere. **a)** For total transmittance measurement the sample is placed at the front opening of the sphere while keeping the other openings closed. **b)** For diffuse reflectance measurement the sample is placed at the back opening of the sphere. For both cases the measurement was repeated for at least 5 different positions across the sample.

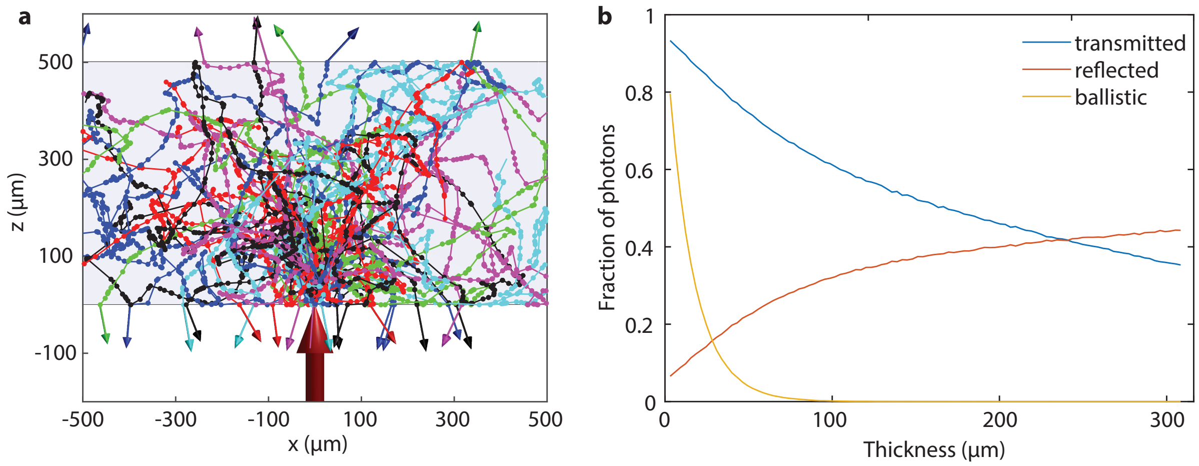

Supplementary Figure 13: Results obtained with Monte-Carlo simulation, illustrating the behavior of light interacting with a scattering medium. The optical parameters chosen in this case were similar to those of the actual samples used in this work ($\mu_s = 63\,\mathrm{mm}^{-1}$ and $g = 0.87$). **a)** Trajectories of 50 photons incident on a 500 µm slab, projected into the x-z plane. **b)** The fractions of transmitted, reflected and ballistic photons as a function of the sample thickness.

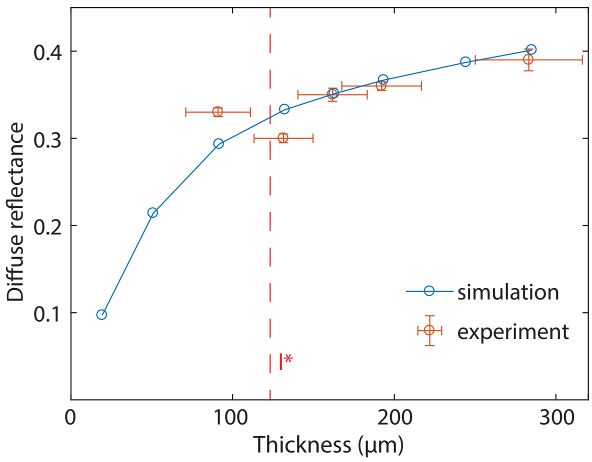

Supplementary Figure 14: Experimental (see Supplementary Fig. 12) and simulated diffuse reflectance values for increasing thickness of phantoms. Monte Carlo simulation used $\mu_s = 63\,\mathrm{mm}^{-1}$ and the phase function ($g = 0.87$) as calculated by Mie theory, taking into account the concentration, size and refractive index of the scattering polystyrene spheres and the refractive index of the surrounding medium. The x-axis error bars indicate the standard error of the phantom thickness, while the y-axis error bars are estimated from multiple measurements at different points on the same phantom. Five different locations across each phantom were used to derive the errors. The experimental and simulation results agree well, validating the values of scattering parameters of the phantoms. The dashed red line denotes the transport length above which light propagation transitions into the diffusive regime.
