# Supplementary material for: Deep tissue localization and sensing using optical microcavity probes
Source: Nat Commun. 2022 Mar 11;13:1269. doi: 10.1038/s41467-022-28904-6 (PMC8917156; doi:10.1038/s41467-022-28904-6)
Supplement: Supplementary file 2 — Description of Additional Supplementary Files [file 41467_2022_28904_MOESM2_ESM.docx]

**Description of Additional Supplementary Files**

**Supplementary Video 1:** The video shows a hyperspectral image of a region with three microcavities under a 1.7 l* thick phantom. Wavelength is changing with time, and the corresponding intensity maps are displayed. Local bursts in intensity appear whenever the wavelength matches a WGM peak of a particular microcavity. At the end of the video, intensity map of the image summed over all wavelengths (i.e. a regular image) is shown and the red circles indicate the reconstructed positions of the microcavities for each spectral peak.
