# Supplementary material for: Deep tissue localization and sensing using optical microcavity probes
Source: Nat Commun. 2022 Mar 11;13:1269. doi: 10.1038/s41467-022-28904-6 (PMC8917156; doi:10.1038/s41467-022-28904-6)
Supplement: Supplementary file 4 — Reporting Summary [file 41467_2022_28904_MOESM4_ESM.pdf]

nature portfolio

Corresponding author(s): Matjaž Humar

Last updated by author(s): Dec 30, 2021

Reporting Summary

Nature Portfolio wishes to improve the reproducibility of the work that we publish. This form provides structure for consistency and transparency in reporting. For further information on Nature Portfolio policies, see our Editorial Policies and the Editorial Policy Checklist.

Statistics

For all statistical analyses, confirm that the following items are present in the figure legend, table legend, main text, or Methods section.

n/a	Confirmed	
☐	☒	The exact sample size (*n*) for each experimental group/condition, given as a discrete number and unit of measurement
☐	☒	A statement on whether measurements were taken from distinct samples or whether the same sample was measured repeatedly
☐	☒	The statistical test(s) used AND whether they are one- or two-sided<br>*Only common tests should be described solely by name; describe more complex techniques in the Methods section.*
☒	☐	A description of all covariates tested
☒	☐	A description of any assumptions or corrections, such as tests of normality and adjustment for multiple comparisons
☐	☒	A full description of the statistical parameters including central tendency (e.g. means) or other basic estimates (e.g. regression coefficient) AND variation (e.g. standard deviation) or associated estimates of uncertainty (e.g. confidence intervals)
☐	☒	For null hypothesis testing, the test statistic (e.g. *F*, *t*, *r*) with confidence intervals, effect sizes, degrees of freedom and *P* value noted<br>*Give P values as exact values whenever suitable.*
☒	☐	For Bayesian analysis, information on the choice of priors and Markov chain Monte Carlo settings
☒	☐	For hierarchical and complex designs, identification of the appropriate level for tests and full reporting of outcomes
☒	☐	Estimates of effect sizes (e.g. Cohen's *d*, Pearson's *r*), indicating how they were calculated

Our web collection on statistics for biologists contains articles on many of the points above.

Software and code

Policy information about availability of computer code

Data collection	Commercial software Andor Solis (v4.32.300000.0) was used to collect all spectra and images.
Data analysis	Custom code written in Matlab (R2019a) was used to analyze all the spectral an image data and to perform Monte Carlo simulations. The code that support the findings of this study are available in Zenodo repository with the identifier doi:10.5281/zenodo.5809094.

For manuscripts utilizing custom algorithms or software that are central to the research but not yet described in published literature, software must be made available to editors and reviewers. We strongly encourage code deposition in a community repository (e.g. GitHub). See the Nature Portfolio guidelines for submitting code & software for further information.

Data

Policy information about availability of data

All manuscripts must include a data availability statement. This statement should provide the following information, where applicable:

- Accession codes, unique identifiers, or web links for publicly available datasets
- A description of any restrictions on data availability
- For clinical datasets or third party data, please ensure that the statement adheres to our policy

The data that support the findings of this study are available in Zenodo repository with the identifier doi:10.5281/zenodo.5809094.

Field-specific reporting

Please select the one below that is the best fit for your research. If you are not sure, read the appropriate sections before making your selection.

- [x] Life sciences
- [ ] Behavioural & social sciences
- [ ] Ecological, evolutionary & environmental sciences

For a reference copy of the document with all sections, see nature.com/documents/nr-reporting-summary-flat.pdf

Life sciences study design

All studies must disclose on these points even when the disclosure is negative.

Sample size	Sample size was not determined in advance. Multiple measurements provided statistics from which it could be deduced that the results are either consistent or statistically different. Usually three to ten measurements were sufficient to verify whether the results are within the measurement inaccuracy and without systematic discrepancies. Different sample sizes are due to different types of experiments (i.e. phantoms, biological tissues, cells, pH and temperature measurements).
Data exclusions	No data were excluded from the analyses.
Replication	Localization and spectral unmixing measurements on non-biological samples were repeated tens of times. Localization and spectral unmixing on biological tissues were performed on at least 15 samples collected from a total of 5 animals. The experiments with cells were reproduced for 8 independent cell cultures. To measure pH and temperature response, a total of 9 and 5 coated microcavities were used, respectively. The reproduced experiments led to the same conclusions within the measurement error. Reproducibility was ensured for all the representative microscope images by repeating the same acquisition for a range of at least 5 different positions on at least 3 distinct samples.
Randomization	Randomization is not relevant to our study because there were no different experimental groups.
Blinding	Blinding is not relevant to our study because there were no different experimental groups.

Reporting for specific materials, systems and methods

We require information from authors about some types of materials, experimental systems and methods used in many studies. Here, indicate whether each material, system or method listed is relevant to your study. If you are not sure if a list item applies to your research, read the appropriate section before selecting a response.

Materials & experimental systems

n/a	Involved in the study	
x		Antibodies
	x	Eukaryotic cell lines
x		Palaeontology and archaeology
x		Animals and other organisms
x		Human research participants
x		Clinical data
x		Dual use research of concern

Methods

n/a	Involved in the study	
x		ChIP-seq
x		Flow cytometry
x		MRI-based neuroimaging

Eukaryotic cell lines

Policy information about cell lines

Cell line source(s)	HeLa cells were kindly provided by the Biophysics laboratory, Department of Condensed Matter Physics, J. Stefan Institute. Original source of HeLa cells was ECACC (European Collection of Cell Cultures).
Authentication	The cell line was not authenticated.
Mycoplasma contamination	Cells were not tested for mycoplasma contamination.
Commonly misidentified lines (See ICLAC register)	None
